# Increased serum levels of betatrophin in pancreatic cancer-associated diabetes

**DOI:** 10.18632/oncotarget.9815

**Published:** 2016-06-03

**Authors:** Hendra Susanto, Ta-Yu Liu, Chang-Chiang Chen, Jerry D.T. Purnomo, Shu-Fan Chen, Chih-Hong Wang

**Affiliations:** ^1^ Department of Biological Science and Technology, National Chiao Tung University, Hsinchu 300, Taiwan; ^2^ Department of Internal Medicine, National Taiwan University Hospital Hsin-Chu Branch, Hsinchu 300, Taiwan; ^3^ Institute of Statistics, National Chiao Tung University, Hsinchu 300, Taiwan

**Keywords:** betatrophin, diabetes, insulin resistance, glucose intolerance, pancreatic cancer

## Abstract

Long-standing diabetes or glucose intolerance is recognized as a crucial event in the process of pancreatic cancer. Betatrophin, a novel liver-derived hormone, promotes β-cell proliferation and improves glucose intolerance. However, the relationship between betatrophin and PDAC-associated diabetes is not fully understood. To evaluate the serum betatrophin levels in PDAC-associated diabetes, a total 105 Taiwanese subjects including 15 healthy subjects, and 12 patients having PDAC with normal glucose tolerance (PDAC-NGT), 12 patients having PC with impaired glucose tolerance (PDAC-IGT), and 66 patients having PC with diabetes mellitus (PDAC-DM) were enrolled for this study. Serum betatrophin and carbohydrate antigen 19-9 (CA19-9) levels were analyzed by enzyme-linked immunosorbent assay (ELISA). Compared to healthy subjects, PDAC patients had higher levels of betatrophin and CA19-9. Consistently, betatrophin protein was significantly expressed in pancreatic ductal of PDAC-associated DM patients using immunohistochemistry (IHC) method. Furthermore, multivariate regression analysis showed the betatrophin was significantly and positively independent with T category (β= 0.605, P=0.010), serum albumin (β= 0. 423, P=0.021), lipase (β= 0.292, P=0.039), and blood urea nitrogen (BUN) (β= 0.303, P=0.040). Further, the betatrophin was three folds of having PDAC-associated diabetes with the highest odds ratio [OR=3.39; 95% CI (1.20–9.57); P=0.021) and receiver operating characteristic (ROC) curve analysis showed that AUC value of betarophin was 0.853 which is slightly larger than AUC value of CA19-9 (0.792) in PDAC-DM patients. Interestingly, AUC value of betarophin plus CA19-9 was 0.988 in PDAC-DM patients. Therefore, betatrophin combined CA19-9 may serve as a potential biomarker for PDAC-associated diabetes.

## INTRODUCTION

Pancreatic ductal adenocarcinoma (PDAC), one of the most aggressive human cancers, is the fourth leading cause of cancer-related deaths in the United States [1]. The incidence and mortality rates of PDAC are similar, with the 5-year survival rate of ~5% [2, 3]. Most PDAC patients are diagnosed at an advanced stages, and only 10% to 20% of patients are resectable at the time of presentation [4]. Based on human epidemiologic and animal studies, most PDAC patients have glucose intolerance [5] and about 80% of PDAC patients are either glycemic or diabetic and this exists in the pre-symptomatic phase [6]. PDAC-associated diabetes can occur at a resectable stage, while resection of pancreatic tumor improves glucose intolerance [7]. Therefore, discovery of novel biomarkers for PDAC-associated diabetes may apply for diagnosis of resectable PDAC at early stages.

Dysfunctions of β-cell and insulin resistance are often seen in PDAC. Recently, betatrophin, a newly recognized liver-derived hormone, has been implicated in glucose metabolism and β-cell proliferation [8]. It significantly and specifically promotes pancreatic β-cell proliferation and improves glucose intolerance in mouse models of insulin resistance [9]. These results suggest betatrophin to be a liver-derived protein that triggers compensatory β-cell proliferation upon insulin resistance [9]. Further, serum levels of betatrophin are positively associated with type 1 diabetes mellitus (T1DM), type 2 diabetes mellitus (T2DM) [10–12], hyperlipidemia [13], and indexes of insulin resistance [14]. Nevertheless, it is unclear whether serum betatrophin levels are correlated with PDAC-associated diabetes and whether serum betarophin levels can serve as a biomarker for PDAC-associated diabetes to apply for diagnosis of resectable PDAC.

The aim of this study is to investigate the relationship between serum betatrophin concentrations and PDAC with or without glucose intolerance, and to determine whether betatrophin levels can serve as a biomarker for PDAC-associated diabetes. We investigated serum betatrophin levels in healthy subjects and PDAC patients with various stages of glucose tolerance: normal glucose tolerance (NGT), impaired glucose tolerance (IGT), and diabetes mellitus (DM). We hypothesized that betatrophin is correlated with PDAC-associated diabetes and can serve as biomarker to predict PDAC-associated diabetes for surgical resection of tumor.

## RESULTS

### Baseline characteristics of the total study participants

**Figure 1:**
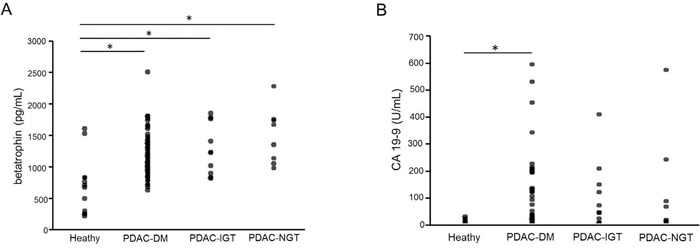
Serum betatrophin and CA19-9 concentrations of healthy and PDAC patients with NGT, IGT, and DM Normal Glucose Tolerance (NGT) [≤ 99 mg/dL (5.5. mmol/L)], Impaired Glucose Tolerance (IGT) [100-125 mg/dl (5.6-6.9 mmol/L)], Diabetes Mellitus (DM) [≥ 126 mg/dl (7 mmol/L)]. Data are means ± SD. *, P<0.05 vs. healthy subjects.

**Figure 2:**
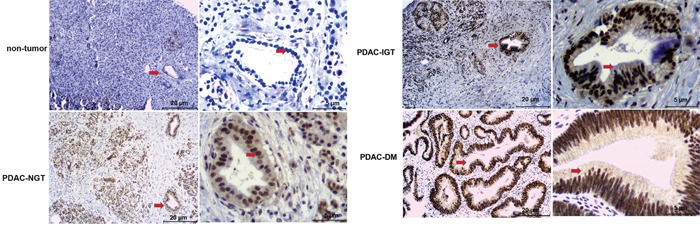
Betatrophin protein expression in human pancreatic cancer cells using immunohistochemical staining Immunohistochemical staining did not show immunoreactivity in the normal pancreas, whereas there was strong immunoreactivity in the pancreatic ductal of pancreatic cancer cells (arrows) in PDAC-NGT, IGT, and DM.

Table 1 summarizes the clinical baseline characteristics of the four groups (healthy control, PDAC-NGT, PDAC-IGT, and PDAC-DM). Compared with healthy control subjects, the serum levels of triglyceride, albumin, alkaline phosphatase (ALP), alanine aminotransferase (ALT), aspartate aminotransferase (AST), amylase, lipase, and blood urea nitrogen (BUN) were significantly increased in PDAC-NGT, PDAC-IGT and PDAC-DM patients, while the hemoglobin and hematocrit (Hct) were significantly decreased. In addition, plasma levels of insulin were significantly increased in PDAC-DM patients. No statistically significant was found in sex and age between healthy controls and PDAC groups. Interestingly, PDAC patients had higher levels of the betatrophin than healthy control subjects. The betatrophin levels from lowest to highest were healthy subjects, PDAC-DM, PDAC-IGT and PDAC-NGT (Table 1 and Figure 1A). Consistently, betatrophin protein was significantly expressed in pancreatic ductal of PC-associated DM patients using immunohistochemistry (IHC) method (Figure 2). These data suggest that higher glucose levels may affect betatrophin expression from liver and adipose tissue in PDAC patients.

**Table 1:** Baseline characteristics of the study population

	Healthy	PDAC-NGT	PDAC-IGT	PDAC-DM
N	15	12	12	66
Age (years)	66.80 ± 1.43	65.87 ± 2.62	67.27 ± 2.44	67.59 ± 0.97
% of male	67	67	58	70
FBG (mg/dL)	101.80 ± 4.78	75.93 ± 6.08^*^	111.38 ± 2.46^†^	205.49 ± 6.39^‡^
Cholesterol (mg/dL)	180.33 ± 11.55	104.83 ± 8.43^*^	108.45 ± 5.11^*^	106.15 ± 3.48^*^
Triglyceride (mg/dlL)	100.93 ± 16.94	161.41 ± 13.32^*^	191.92 ± 15.19^*^	181.46 ± 7.86^*^
Hemoglobin (%)	14.45 ± 0.48	11.17 ± 0.59^*^	12.80 ± 0.34^*^	12.13 ± 0.17^*^
Albumin (g/dL)	4.87 ± 0.23	3.26 ± 0.20^*^	3.87 ± 0.12^*^	3.61 ± 0.07^*^
ALP (U/L)	107.53 ± 23.86	297.95 ± 69.99^*^	236.16 ± 51.04^*^	315.55 ± 24.77^*^
ALT (U/L)	9.80 ± 1.80	106.00 ± 25.84^*^	181.83 ± 32.54^*^^†^	190.55 ± 23.64^*^^†^
AST (U/L)	9.15 ± 1.44	81.77 ± 10.42^*^	66.72 ± 18.98^*^	153.24 ± 17.48^*^^†^
Amylase (U/L)	28.67 ± 2.15	269.33 ±116.31^*^	176.22 ± 68.62^*^	263.00 ± 57.22^*^
Lipase (U/L)	20.73 ± 1.80	817.71 ± 532.71^*^	499.80 ± 231.88^*^	532.72 ± 83.68^*^
Survival (years)		1.53 ± 0.32	0.58 ± 0.11^*^	0.46 ± 0.17^*^
Hct (%)	45.85 ± 3.57	27.16 ± 5.34^*^	29.05 ± 4.01^*^	32.12 ± 1.39^*^^†^
BUN (mg/dL)	4.24 ± 0.62	14.78 ± 2.64	15.33 ± 1.19	15.72 ± 0.89
Creatinitne (μmol/L)	79.12 ± 12.05	82.54 ± 13.15	95.45 ± 9.29	87.82 ± 15.34
T category (I&II : III&IV)		8 : 4	6 : 6	38 : 28
N category (N0 : N1)		2 : 10	7 : 5	42 : 24
insulin (mU/L)	2.25 ± 0.41	2.45 ± 0.77	4.08 ± 1.57	5.48 ± 0.63^*^
CA 19-9 (U/mL)	9.39 ± 2.75	67.09 ± 36.53	88.94 ± 29.02	88.54 ± 16.19^*^
betatrophin (pg/mL)	702.51 ± 112.78	1398.13 ±15 7.07^*^	1315.77 ± 119.54^*^^†^	1205.79 ± 44.44^*^^†^^‡^

Abbreviations: FBG: Fasting Blood Glucose; ALP: Alkaline Phosphatase; ALT: Alanine Aminotransferase; AST: Aspartate Aminotransferase; Hct: hematocrit; BUN: blood urea nitrogen. Normal Glucose Tolerance (NGT) [≤ 99 mg/dL (5.5. mmol/L)], Impaired Glucose Tolerance (IGT) [100-125 mg/dl (5.6-6.9 mmol/L)], Diabetes Mellitus (DM) [≥ 126 mg/dl (7 mmol/L)]. Data are means ± SD.

* P<0.05 vs. healthy subjects;

† P<0.05 vs. PaC-NGT;

‡ P<0.05 vs. PaC-IGT. TNM classification is according to the International Union against Cancer (UICC, 2002).

Moreover, serum carbohydrate antigen 19-9 (CA19-9) is commonly used biomarker in pancreatic cancer [15]. We found that CA19-9 was significantly increased in PDAC-DM patients as compared with healthy subjects (Table 1 and Figure 1B). Therefore, both serum levels of betatrophin and CA19-9 were significantly increased in PDAC-DM patients.

### Univariate correlations in the total sample

The serum betatrophin levels were found to be positively correlated with albumin, amylase, lipase, BUN, and T category (P< 0.05; Table 2), while its levels were inversely correlated with cholesterol, hemoglobin, and Hct (P< 0.05; Table 2). Interestingly, although betatrophin was not correlated with fasting blood glucose (FBG) in all subjects, it was significantly associated with PDAC patients with various stages of glucose tolerance (PDAC-NGT [*r*= −0.589; P=0.015]; PDAC-IGT [*r*= −0.554; P=0.017]; PDAC-DM [*r*=−0.586; P=0.002], respectively).

**Table 2:** Univariate correlation with serum betatrophin levels in all participants, healthy, PDAC with NGT, IGT, and DM groups

	All subjects		Healthy		PDAC-NGT		PDAC-IGT		PDAC-DM	
	*r*	*P*	*r*	*P*	*r*	*P*	*r*	*P*	*r*	*P*
FBG (mg/dL)	−0.091	0.380	−0.010	0.971	−0.589	0.015^*^	−0.554	0.017^*^	−0.586	0.002^*^
Cholesterol (mg/dL)	−0.358	<0.001^*^	0.112	0.692	0.408	0.031^*^	0.548	0.018^*^	−0.309	0.015^*^
Triglyceride (mg/dlL)	0.131	0.207	−0.086	0.761	0.621	0.010^*^	0.145	0.671	−0.172	0.186
Hemoglobin (%)	−0.205	0.046^*^	−0.088	0.755	0.299	0.472	0.243	0.472	0.019	0.882
Albumin (g/dL)	0.321	0.002^*^	0.320	0.244	−0.264	0.075	0.388	0.048^*^	−0.299	0.044^*^
ALP (U/L)	0.122	0.243	−0.195	0.486	0.025^*^	0.028^*^	−0.111	0.744	−0.171	0.190
ALT (U/L)	0.026	0.800	0.236	0.396	−0.418	0.034^*^	0.107	0.755	−0.172	0.186
AST (U/L)	−0.065	0.535	0.111	0.694	0.048	0.910	−0.211	0.558	−0.297	0.020^*^
Amylase (U/L)	0.228	0.045^*^	0.092	0.745	0.623	0.002^*^	0.292	0.045^*^	0.112	0.439
Lipase (U/L)	0.441	<0.001^*^	0.019	0.947	0.390	0.049^*^	0.680	0.004^*^	0.350	0.011^*^
Hct (%)	−0.270	0.010^*^	0.336	0.220	0.370	0.058	0.830	0.003^*^	0.155	0.242
BUN (mg/dL)	0.310	0.002^*^	0.122	0.665	0.254	0.089	0.130	0.704	−0.016	0.905
Creatinine (μmol/L)	0.162	0.118	−0.350	0.201	0.447	0.045^*^	0.310	0.075	0.165	0.203
T category	0.423	<0.001^*^	-	-	0.689	0.005^*^	0.351	0.050^*^	0.154	0.237
N category	0.198	0.083	-	-	0.333	0.071	0.245	0.122	0.196	0.133

Abbreviations: FBG: Fasting Blood Glucose; ALP: Alkaline Phosphatase; ALT: Alanine Aminotransferase; AST: Aspartate Aminotransferase; Hct: hematocrit; BUN: blood urea nitrogen. Normal Glucose Tolerance (NGT) [≤ 99 mg/dL (5.5. mmol/L)], Impaired Glucose Tolerance (IGT) [100-125 mg/dl (5.6-6.9 mmol/L)], Diabetes Mellitus (DM) [≥ 126 mg/dl (7 mmol/L)].

* p<0.05 is considered significant. TNM classification is according to the International Union against Cancer (UICC, 2002). Correlations were estimated by spearman correction test.

### Multivariate regression analysis in the total sample

To verify independent associations, multiple linear regression analysis was performed. Here, T category of PDAC was a positive, independent and significant predictor of serum betatrophin levels (β= 0.605, P=0.010; Table 3). Furthermore, the serum CA19-9 (β= 0. 001, P=0.047), albumin (β= 0. 423, P=0.021), lipase (β= 0.292, P=0.039), and BUN (β= 0.303, P=0.040) were also significantly and positively associated with serum betatrophin levels (Table 3).

**Table 3:** Univariate correlations and multivariate regression analysis with serum betatrophin levels

Independent Variables	Univariate analysis		Multivariate analysis	
	*r*	*P*	*β*	*P*
FBG (mg/dL)	−0.091	0.380		
CA19-9	0.386	0.001^*^	0.001	0.047
Cholesterol (mg/dL)	−0.358	<0.001^*^	−0.103	0.475
Triglyceride (mg/dlL)	0.131	0.207		
Hemoglobin (%)	−0.205	0.046^*^	−0.084	0.567
Albumin (g/dL)	0.321	0.002^*^	0.423	0.021^*^
ALP (U/L)	0.122	0.243		
ALT (U/L)	0.026	0.800		
AST (U/L)	−0.065	0.535		
Amylase (U/L)	0.228	0.045^*^	0.084	0.465
Lipase (U/L)	0.441	<0.001^*^	0.292	0.039^*^
Survival (years)	0.114	0.256		
Hct (%)	−0.270	0.010^*^	0.319	0.150
BUN (mg/dL)	0.310	0.002^*^	0.303	0.040^*^
Creatinine (umol/L)	0.162	0.118		
T category	0.423	<0.001^*^	0.605	0.010^*^
N category	0.198	0.083		

Abbreviations: FBG: Fasting Blood Glucose; ALP: Alkaline Phosphatase; ALT: Alanine Aminotransferase; AST: Aspartate Aminotransferase; Hct: hematocrit; BUN: blood urea nitrogen. TNM classification is according to the International Union against Cancer (UICC, 2002). Multivariate regression analysis between betatrophin (dependent variable) and the independent variables shown. Standardized β coefficients and P values are given.

* p<0.05 is considered significant.

### Association between serum betatrophin levels and PDAC patients with various stages of glucose tolerance

**Figure 3:**
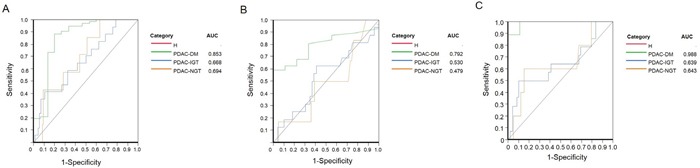
Receiver Operating Characterictic (ROC) curve analysis for betatrophin, CA 19-9, and betatrophin combined CA19-9 in pancreatic cancer-associated with diabetes (PC-DM), impaired glucose tolerance (PC-IGT), and non-diabetes (PC-NGT) **A.** ROC curve analysis for betatrophin in PC-DM and found that AUC_betatrophin_ is 0.853; **B.** ROC curve analysis for CA19-9 in PC-DM and found that AUC_CA19-9_ is 0.792; **C.** ROC curve analysis for betatrophin combined CA19-9 in PC-DM and found that AUC_betatrophin-CA19-9_ is 0.988.

We used the ordinal logistic regression analysis to estimated associations between serum betatrophin levels and PDAC patients with various stages of glucose tolerance (NGT: FBG < 99 mg/dL. IGT: FBG between 100 mg/dL and 125 mg/dL. DM: FBG >126 mg/dL). We found that betatrophin [OR= 3.39, 95% CI (1.20-9.57), P=0.021] was higher than CA 19-9 [OR= 1.00, 95% CI (0.09-1.01), P=0.295], amylase [OR= 1.01, 95% CI (1.00-1.02), P=0.002], and lipase [OR= 1.01, 95% CI (1.00-1.01), P=0.151] (Table 4). Moreover, we used receiver operating characteristic (ROC) analysis to assess the potential benefit of using betatrophin instead of CA19-9. When we compared the area under the curve (AUC) value of each biomarker, the sensitivity and specificity of betatrophin was significantly higher than CA 19-9 (Figure 3). AUC value of betatrophin in PDAC-DM patients (0.853) was higher than PDAC-IGT (0.668) and PDAC-NGT (0.694), while AUC value of CA19-9 in PDAC-DM patients (0.792) was higher than PDAC-IGT (0.530) and PDAC-NGT (0.479). Interestingly, betatrophin combined CA 19-9 showed a significant predict PDAC-associated diabetes and AUC value of betatrophin combined CA19-9 in PDAC-DM (0.9877) was higher than in PDAC-IGT (0.639), and PDAC-NGT (0.643) (Figure 3). Therefore, the betatrophin combined CA19-9 may serve as a potential biomarker for PDAC-associated diabetes.

**Table 4:** Relationships between serum betatrophin levels and pancreatic cancer with various stages of glucose tolerance

Covariates	OR (95% CI)	P-value
Betatrophin	3.39 (1.20 - 9.57)	0.021^*^
Amylase	1.01 (1.00 - 1.02)	0.002^*^
Lipase	1.00 (1.00 - 1.01)	0.151
CA 19-9	1.00 (0.09 - 1.01)	0.295
Cholesterol	0.97 (0.95 - 0.98)	<0.001^*^
Triglyceride	1.01 (1.01 - 1.02)	0.002^*^
Hemoglobin	0.63 (0.48 - 0.82)	0.001*
ALP	1.01 (1.00 - 1.01)	<0.001^*^
ALT	1.01 (1.00 - 1.01)	0.008^*^
AST	1.02 (1.01 - 1.03)	<0.001^*^

Abbreviations: ALP: Alkaline Phosphatase; ALT: Alanine Aminotransferase; AST: Aspartate Aminotransferase.

Insulin resistance classification: [Fasting Blood Glucose: Normal Glucose Tolerance (NGT) [≤ 99 mg/dl (5.5. mmol/L)], Impaired Glucose Tolerance (IGT) [100-125 mg/dl (5.6-6.9 mmol/L)], and Diabetes Mellitus (DM) [≥ 126 mg/dl (7 mmol/L). Ordinal logistic regression analysis between insulin resistance category (dependent variable) and the independent variables shown. *P* values and OR (95% CI) are given.

* Significant with *p* < 0.05.

## DISCUSSION

PDAC is the fourth most common cause of death from cancer in the western world, and long-term survival remains poor with a 5-year survival rate less 5% [16]. Resection is associated with improved survival but this is only possible in approximately 10% of patients [7]. PDAC patients often have impaired glucose tolerance or diabetes at early stage, while surgical resection of the tumor improves glucose tolerance and insulin resistance [7, 17]. The development of PDAC-DM is a promising clue for the early detection of pancreatic cancer, but how PDAC causes diabetes is largely unknown. We analyzed that serum betatrophin levels in patients with PDAC-NGT, PDAC-IGT, and PDAC-DM as well as healthy subjects as control. Our data indicated that betatrophin levels not only predicted PDAC-DM, but also served as biomarker for tumor stages in PDAC.

The pathogenesis of PDAC-associated diabetes and its biochemical mediators are not known. This is unlikely to be simply due to destruction of the gland by the tumor. The high prevalence of glucose intolerance or diabetes occurs at early stage of PDAC and the tumor size is smaller than 2 cm that provide an ideal clue for resection of tumors to increase survival [18]. However, PDAC-DM cannot be easily identified from other types of diabetes by clinical signs and symptoms, and specific markers are urgently needed [19]. Several factors related to PDAC-DM have been identified, such as insulin and C-peptide [6, 20, 21]. Insulin and C-peptide levels in diabetes are higher than in healthy controls, while low levels of insulin and C-peptide are seen in PDAC-associated diabetes [22]. This discrepancy could be simply due to decrease of pancreatic β-cell mass in PDAC-associated diabetes. However, Michaud et al. studies showed that fasting insulin and C-peptide were not related to PDAC, while they observed a slight linear association for nonfasting C-peptide and PDAC [22]. Therefore, the role of insulin and C-peptide levels and other factors in the early diagnosis of PDAC-DM remains unclear.

Recently, betatrophin has been recognized as a novel hormone from liver and adipose tissue, and promotor of β-cell mass proliferation and improved glucose intolerance in mouse models of insulin resistance [8, 9]. Circulating betatrophin levels have been reported to correlate with T1D [11] and T2D [23]. Further, betatrophin is also called hepatocellular carcinoma (HCC)-associated Gene TD26, which is highly expressed in HCC [24, 25]. Several studies have shown that liver and pancreas arise from a common multipotent population of endoderm and exogenous glucocorticoid can cause the conversion of pancreatic exocrine cells into hepatocytes [26–28]. We therefore hypothesized that betatrophin is correlated with PDAC-associated diabetes and can serve as biomarker to predict PDAC-associated diabetes for surgical resection of tumor. In the present study, we observed that serum betatrophin levels were significantly increased in PDAC-NGT, PDAC-IGT, and PDAC-DM patients as compared with healthy subjects. This may be caused by decrease of pancreatic β-cell mass in PDAC patients, which in turn cause increased betatrophin expression to promote β-cell mass proliferation. However, although serum betatrophin levels were higher in PDAC patients than in healthy subjects, its levels in PDAC-associated glucose intolerance or diabetes were lower than in PDAC patients with normal glucose tolerance. It has been known that betatrophin is not only produced by liver, but also is secreted by adipose tissue [29]. The possibility is that elevated serum betatrophin levels is due to cancer-induced anorexia at advanced stages. The early stages of PDAC appear PDAC-associated glucose intolerance or diabetes. Indeed, Barja-Fernández et al. studies have demonstrated that circulating betatrophin levels were significantly elevated in anorexic patients, whereas its levels were reduced in morbidly obese women [29]. However, future studies are needed to investigate this speculation.

Furthermore, we also demonstrated that betatrophin was strongly associated with PDAC-associated diabetes. The betatrophin was three folds of having PDAC-associated diabetes with the highest odds ratio [OR=3.39; 95% CI (1.20–9.57); P=0.021). The ROC analysis showed that AUC value of betarophin was 0.853. Serum carbohydrate antigen 19-9 (CA19-9) is commonly used biomarker in pancreatic cancer [15]. However, the precise knowledge of CA19-9 in pancreatic cancer diagnosis, staging, determining resectability, response to chemotherapy and prognosis remains limited. In our study, we also measured CA19-9, and found that the AUC value of CA 19-9 in PDAC-DM was 0.792 that is lower than that of betatrophin. Interestingly, AUC value of betatrophin combined CA19-9 was 0.988 in PDAC-DM patients. Therefore, betatrophin combined CA19-9 may serve as a potential biomarker for PDAC-associated diabetes.

A number of studies have showed that betatrophin levels were correlated with T1D and T2D and increased in T1D and T2D patients [11, 12, 23, 30]. This increase may be related to increased insulin resistance and higher demand for insulin in T1D and T2D patients. Although the mechanisms underlying increased betatrophin levels in T1D and T2DM patients remain elusive, it is possible that liver and pancreas arise from a common multipotent population of endoderm and couple with the increased β-cell proliferation rate response to tissue-specific insulin resistance in the liver. Recently, Yi et al. [9] studies reported that hepatic expression of betatrophin was upregulated in mouse models of insulin resistance in which β-cell proliferation is increased. This model induced insulin resistance results in dramatic pancreatic β-cell proliferation. Indeed, overexpression of betatrophin by using adenovirus mediated method caused β-cell expansion and enhanced glucose clearance. These results suggest betatrophin to be a liver-derived protein that triggers compensatory β-cell proliferation upon insulin resistance. However, Gusarova et al. reported that overexpression of betatrophin in liver or knockout of betatrophin in mice does not alter beta cell expansion nor glucose metabolism when mice kept on a high fat diet (HFD) for 8 weeks [31]. Although this report provides contradictory evidence for a physiological role of betatrophin in the mouse β-cell function, the conclusion is far from conclusive. The normal glucose tolerance and glucose levels of the betatrophin KO mice suggest that betatrophin is not required physiologically for the maintenance of the β-cell mass. However, betatrophin (Angptl8) is an angiopoietin-like proteins family and 90% homologous to Angptl3 and Angptl4 genes. Thus, it is possible to obscure the phenotype of the betatrophin KO mice due to a compensatory increase in homologous genes Angptl3 and Angptl4 levels. Indeed, Zhang et al. used the same insulin resistance model and found that betatrophin was significantly induced, whereas Angptl4 was suppressed [32]. Therefore, we speculate that the increase in serum betatrophin in PDAC-associated diabetes might be attributable to a defensive response, which may represent an ability to adapt to PDAC resulting in decline of β-cell proliferation rate and increased blood glucose concentrations. Indeed, plasma insulin levels of PDAC-DM patients were higher than in healthy subjects.

The present research study had a number of limitations. First, this study is limited by its cross-sectional design and provides no temporal interpretation of reported associations. Future studies should longitudinally evaluate the association between betatrophin levels and PDAC-associated diabetes to better understand how betatrophin could alter normal glucose metabolism in PDAC patients and predict of PDAC-associated diabetes. Second, serum betatrophin levels were not an end point of recruited PDAC patients in the study, and measurements of serum betatrophin levels were made on stored samples, although the samples were relatively fresh. Third, our study is based on single measurement of serum betatrophin, which may not reflect betatrophin levels over time. Thus, serial changes in serum betatrophin in PDAC patients need to be measured to further clarify the role of betatrophin in the pathogenesis of PDAC-associated diabetes.

In summary, our results indicate for the first time that serum betatrophin levels were significantly correlated with PDAC-associated diabetes. The serum levels of betatrophin combined CA19-9 may serve as a potent biomarker for the early diagnosis of PDAC. Future studies are required to address the role of betatrophin in the pathogenesis of PDAC-associated diabetes.

## MATERIALS AND METHODS

### Study population

This case control study was approved by the institutional review board of the National Chiao Tung University, Taiwan. Eligible subjects were defined as 90 patients with PDAC who were admitted to the Taipei Veterans General Hospital, Taipei, Taiwan, between 1st January 2005 and 31st December 2012. The study protocol was performed according to the principles of the Declaration of Helsinki, and the written informed consent was obtained from all participants. Total 105 Taiwanese subjects are involved in this study. In brief, 15 healthy control subjects, and 12 patients having PDAC with normal glucose tolerance (PDAC-NGT), 12 patients having PDAC with impaired glucose tolerance (PDAC-IGT), and 66 patients having PDAC with diabetes mellitus (PDAC-DM) were enrolled for this study. Abdomen computerized tomography (CT) scan was used to confirm the patients with suspected for PDAC. Patients and controls were age from 60 to 75 years old. In addition, patients related to alcohol and tobacco consumption (e.g., respiratory diseases, peptic ulcer and hepatic disease) and had concurrent cancer at another organ site or past history of cancer were excluded. The control subjects were selected as subjects without disease and not taking any medications. Exclusion criteria were as follows: 1) any evidence of active infection (e.g. fever, or leukocytosis); 2) any evidence of impaired renal, hepatic, or hematopoietic function; 3) no known history of chronic systemic diseases, such as diabetes and hypertension; 4) no long-term medical treatment for chronic systemic diseases; 5) blood tests showing abnormal glucose levels. Moreover, normal glucose tolerance, impaired glucose tolerance and diabetes were diagnosed following the American Diabetes Association criteria: the study subjects were defined as having NGT if fasting blood glucose (FBG) value under 99 mg/dL is NGT. IGT is between 100 mg/dL and 125 mg/dL, and study subjects were defined as having diabetes if FBG value was 126 mg/dL or greater.

### Laboratory analysis

Blood samples were collected after overnight fasting, and serum was stored at minus 20°C. Serum variables were analyzed at the department of medical and chemical laboratory using routine procedures. The serum levels of betatrophin were quantified using a commercially available Enzyme Linked Immunosorbent Assay (ELISA) kit (Wuhan Eiaab Science, Wuhan, China; catalogue No. E11644h) according to the manufacturer's instructions [33]. Current ELISA kit was validated against other available kits showing correlation coefficient of 0.992. Furthermore, the serum levels of CA 19-9 and insulin were quantified using ELISA) kit (IBL America, catalogue No. IB19124, Minneapolis, USA) and Human Insulin ELISA kit (Mercodia, Catalogue No. 10-1113-01, Sweden).

### Biochemical analyses

Fasting glucose concentrations were measured by hexokinase method, triglyceride was assayed using standard enzymic method, and cholesterol was examined by cholesterol oxidase method. Moreover, albumin was determined by immuno-turbidmetric method. Creatinine was measured by Jaffe Reaction Method. All biochemical measurements were performed by Hitachi 7180E automatic biochemical analyzer (Hitachi Instruments Service, Tokyo, Japan). In addition, serum amylase/lipase values were determined (LX-20; Beckman Coulter Fullerton, CA).

### Immunohistochemistry of betatrophin

Immunohistochemistry of betatrophin was performed using surgical human specimens of PDAC. Briefly, paraffin sections were deparaffinized and incubated with methanol containing 0.3% hydrogen peroxide for 15 min, then 10% normal goat serum (Jackson ImmunoResearch Inc., West Grove, PA, USA) was added to the sections to block non-specific staining. The sections were incubated with an anti-human betatrophin rabbit polyclonal antibody (1:400 dilution, GeneTex International Corporation, Hsinchu City, Taiwan) for 1 h at room temperature. After washing with PBS, sections were also incubated with the secondary antibody (rabbit IgG conjugated with horseradish peroxidase, (Jackson ImmunoResearch Inc., West Grove, PA, USA) for 40 min at room temperature. Sections were visualized by immersion in DAB (3,3 diaminobenzidine, Jackson ImmunoResearch Inc., West Grove, PA, USA) as a chromogen. Then nuclear staining was performed using hematoxylin, and each section was embedded.

### Statistical analysis

All statistical analyses were performed using SPSS Software version 20.0 (IBM, Chicago, IL, USA). Differences in serum levels of betatrophin in healthy control subjects, PDAC-NGT, PDAC-IGT, and PDAC-DM patients were assessed by parametric one-way analysis of variance (ANOVA) with tukey post hoc test. Univariate correlations were performed using non-parametric Spearman's correlation test. Afterward, multivariate linear regression analysis was performed to identify independent relationships. Before univariate and multivariate analyses were calculated, the distribution of the respective variables was tested for normality using a Kolmogorov - Smirnov test. Univariate correlations were performed using parametric Pearson ‘s product moment method. Ordinal logistic regression analysis was performed to identify independent relationships. Area under the curve (AUC) were analyzed by ordinal logistic regression receiver operating characteristic (ROC) method with JMP 7.0 software (SAS Institute Inc, NC, USA). A P value less than 0.05 was considered statistically significant.
